# Lung cancer screening care pathways in community settings: An examination of three healthcare systems

**DOI:** 10.1093/annalsats/aaoag029

**Published:** 2026-06-01

**Authors:** Casey Walsh, Lorella Palazzo, Laurel Hansell, Tinnie Louie, Stewart Long, Meagan Brown, Daniel S. Hippe, Gloria D. Coronado, Katie DeCell, Richard J Leone, Saba Lodhi, Nicholas Wysham, Karen J. Wernli, Matthew Triplette

**Affiliations:** 1 Public Health Sciences Division, Fred Hutchinson Cancer Center, Seattle, WA, USA; 2 Kaiser Permanente Washington Health Research Institute, Seattle WA, USA; 3 Clinical Research Division, Fred Hutchinson Cancer Center, Seattle, WA, USA; 4 Population Sciences, University of Arizona Cancer Center, Tucson, AZ, USA; 5 Division of Hematology and Oncology, University of Washington, Seattle, WA, USA; 6 Skagit Regional Health, Mount Vernon, WA, USA; 7 Confluence Health, Wenatchee, WA; 8 The Vancouver Clinic, Vancouver, WA, USA; 9 Washington State University College of Medicine, Spokane, WA, USA; 10 Division of Pulmonary, Critical Care and Sleep Medicine, University of Washington, Seattle, WA, USA

**Keywords:** adherence, community setting, lung cancer screening, patient navigation

## Abstract

**Background:**

Lung cancer screening (LCS) with low-dose computed tomography (LDCT) is recommended for individuals ages 50–80 with a high-risk tobacco history, but implementation of LCS in community settings remains a significant challenge. The benefits of LCS are tempered by both low uptake and low adherence to recommended follow-up, supporting the need for community-engaged research in this area.

**Objective:**

The objective of this study was to understand LCS workflows throughout the care continuum in representative community-care settings.

**Design:**

This is a case study informed by multi-method data collection to characterize three community-based LCS programs in Washington state who are participating in a hybrid effectiveness-implementation trial to enhance LCS program care coordination.

**Participants:**

This research was conducted in collaboration with three community-based LCS referral programs. Participants included program partners who participated in formalized site visits and interviews.

**Approach:**

To develop and refine LCS workflows, we triangulated data from rapid ethnographic assessment site visits (n=5), semi-structured interviews with care providers (n=15), and member checking with key programmatic partners from each site. Rapid Group Analysis Process was used to integrate findings and guide the development and visualization of LCS workflows.

**Key Results:**

The three community-based programs provide LCS services for their regional primary care networks with various levels of centralized programmatic support. LCS workflows from each site demonstrate varied staff involvement and resources along the LCS care continuum. Provider interviews identified the need for patient education and outreach, provider support and resources, and attention to gaps in care along the LCS continuum.

**Conclusions:**

The LCS system-level workflows demonstrate three approaches to LCS care in community settings. LCS workflows can enable the timely identification of barriers and facilitators to improving LCS implementation in community settings.

## Background

Lung cancer remains the leading cause of cancer death in the United States, with >80% of all US cases secondary to cigarette smoking.^1,2^ Lung cancer screening (LCS) with low-dose chest CT (LDCT) can reduce lung cancer mortality by approximately 20% in a high-risk eligible population and has been recommended by the United States Preventive Services Task Force since 2013.^3,4^ Despite >10 years of guideline recommendations, research suggests poor implementation along the LCS care continuum.^5^

Studies have demonstrated there are multiple and multi-level barriers to LCS among both patients and providers.^6^ To support LCS in the face of patient and provider barriers, many health systems have aligned LCS program structures with locally available resources to enhance screening care completion.^7–9^ In the literature, LCS programs have often been summarized as either centralized or decentralized.^10^ Centralized programs are described as those in which patients are referred to a central program or support providers to perform key LCS functions and care coordination throughout the continuum. Decentralized programs offer LDCT screening but care pathways provide little to no programmatic support for LCS beyond usual diagnostic imaging processes. Hybrid programs—ones which provide some features of centralized and programmatic support—may be the most common but are poorly described.^11^

In general, granular care processes within LCS programs are under-reported. System-level care processes likely have a substantial influence on LCS outcomes across the continuum, as centralized programs have consistently been shown to have higher rates of annual follow-up than those classified as decentralized.^12^ Therefore, the objective of this study was to use a multi-method approach to granularly examine the workflows of three community-based LCS referral programs across the LCS care continuum to understand usual care processes in these settings.

## Methods

This study is nested within a larger trial (NCT06324110) which seeks to understand determinants of LCS care outcomes in community-settings and implement system-level interventions that support adherence to LCS follow-up care.^13^ In this study and in the larger trial, the Consolidated Framework for Implementation Research (CFIR) was used to organize data about system-, provider-, and patient-level facilitators and barriers to LCS.^14^ The CFIR domains help to facilitate systematic evaluation of relevant implementation factors while adapting and testing LCS care coordination in new clinical settings. This study was approved by the Fred Hutchinson Cancer Center IRB with a waiver of written informed consent for participants. The objective of this study was to thoroughly describe and summarize LCS care processes for community-based programs in their baseline state prior to intervention deployment (Fall 2024). The participating study sites were Confluence Health, Skagit Regional Health and The Vancouver Clinic. Confluence Health (hereafter referred to as Site A) serves as the major medical provider in north central Washington and is located in Wenatchee, WA. The system provides care to 250,000 patients annually, operates 12 primary clinics, and is a screening catchment center for the region. Skagit Regional Health (Site B) is located in Mt. Vernon, WA. The system provides care to 280,000 patients annually in north-western Washington and has 26 affiliated primary care clinics. The Vancouver Clinic (site C) is a multispecialty medical practice in southwestern WA which includes 24 affiliated primary care clinic sites, serving approximately 150,000 patients annually. All 3 systems use an integrated Epic systems-based EHR.^15^ Sites A and B are intervention study sites where the care coordination intervention is being deployed. Site C is a comparison study site, therefore data collection at Site C did not include provider interviews.

A multi-method approach was used to inform the summarization and visualization of LCS workflows at each study site including rapid ethnographic assessment (REA) virtual and in-person site visits, provider/program partner interviews, and member checking with LCS advisory board members from each site.

### REA site visits

REA is an applied qualitative research method rooted in ethnography and adapted to accommodate shortened timelines and a team-based approach.^16,17^ REAs can be used to understand implementation processes and capture contextual details to improve the design and implementation of interventions.^18^ REA site visits were completed at each of the study sites in 2024, as described below, to gain a deeper understanding of current LCS workflows, as organized by Consolidated Framework for Implementation Research (CFIR) domains.^14^ To plan each visit, we coordinated with participating sites and used purposive sampling to identify individuals from varied disciplines and roles along the LCS continuum. REA visits were led by three team members (LH, LP, MB) with advanced training and expertise in qualitative and ethnographic methods applied to clinical settings. All site visits were facilitated by a semi-structured fieldnote template developed and iteratively refined by the research team and operationalized to the CFIR domains. Data collected during REAs included meeting recordings and notes which were summarized into a standardized observation template. The study team did review LCS materials (such as information handouts and order sets) at these visits, but sites did not have pre-existing LCS workflow documentation.

#### Site A:

There were two REA site visits. The first site visit was a single-day visit conducted virtually through Microsoft Teams. Meetings were held with administrative staff and multi-disciplinary clinical staff.

The second REA site visit was conducted in-person over two days. On the first day, meetings were held with research and administrative staff. An observation was conducted in the radiology department. There was a virtual call with members of the secondary screening site. There was an in-person meeting held with the LCS physician champion. On the second day, team members observed LCS visits that occurred at two affiliated clinic locations.

#### Site B:

There were two site visits. The first visit was conducted virtually via Microsoft Teams and involved multi-disciplinary clinical and administrative staff. The second site visit was conducted in-person over two days. Study team members spoke with providers and clinic staff during meetings, interviews, and observations. Observations were completed of the primary care clinical operations at a representative primary care clinic including a visit encounter with someone eligible for LCS, how to find health maintenance gaps in the electronic health record, and observations of LDCTs being performed at the main medical center.

#### Site C:

Study team members conducted a single virtual site visit, followed by two virtual follow-up meetings to confirm program activities. The site visit meeting involved multi-disciplinary clinical staff, administrators, and support staff. The follow-up meetings involved the LCS program director and a medical assistant from the LCS program. The REA team drafted an initial LCS workflow visualization that was later verified and refined with input from the program LCS navigator and medical director.

### Provider interviews

To better understand the LCS workflows and facilitators and barriers to LCS, study team members conducted semi-structured virtual interviews with key healthcare providers (n=15) from the two study sites which ultimately implemented care coordination interventions (n= 9 from Site A and n=6 from Site B). Provider disciplines included physicians (from pulmonology [n = 1], internal medicine [n = 8], and family medicine [n = 2]), physician assistants (from primary care [n = 2] and family medicine [n =1]), and a nurse practitioner (from family medicine [n = 1]). Interviewees provided verbal consent and were each compensated $40 for their time. Interview prompts explored the provider role(s) and their time at the organization, LCS knowledge and involvement in LCS in their practice, perceptions of importance of LCS and prioritization of LCS during clinic visits, strengths of LCS processes, gaps/challenges in implementing LCS, and LCS processes at the institution (e.g., communication among team members and with patients along the LCS continuum). Each interview lasted approximately 1 hour and was recorded and professionally transcribed.

### LCS advisory board meetings

Advisory board members representing study partners at each site reviewed the current state LCS workflow map from each site to help ensure an accurate representation of current LCS processes. Members included invited local partners relevant to the LCS process including diverse representation from clinical medicine (primary care, radiology, pulmonary care and thoracic surgery), clinical informatics/analytics, research, and administration and supportive services (such as medical assistants, nursing, tobacco cessation staff). Advisory board meetings occur monthly with each site and are held virtually via videoconference, with representation from 5–8 advisory board members and members of the Fred Hutch research team (program manager, principal investigator, research coordinator, staff scientist).

### Analysis

Multi-disciplinary team members (KJW, LP, LH, MT, MB, SL, TL) participated in Rapid Group Analysis Process (Rap-GAP) to analyze the qualitative data collected from interviews.^19^ Prior to the Rap-GAP analysis, team members completed a comprehensive data matrix by site from the REA visits. REA findings, CFIR domains, and other domains of interest relevant to LCS workflows, were used to help inform selection of the group process domains for the interview analysis. Coding memos from the interviews were organized by the Rap-GAP domains and served to synthesize within and across case analysis to identify unique site characteristics and overarching themes. For this analysis, we examined the data directly related to questions about LCS workflow functions. The initial LCS workflow visualizations were developed using the stages of the LCS continuum (e.g., identification, SDM/Ordering) and staff member involvement (e.g., PCP, radiology department) along the LCS continuum, and tailored for each site based on REA data and advisory member feedback.

Data from provider interviews was organized in a master Excel file for each site (provider interviews at Site A and provider interviews at Site B). Worksheet tabs were created for each CFIR-informed and inductive domain within each file. Data within each worksheet tab included the author (e.g., coder), content focus (e.g., provider awareness of LCS), and representative quotes. Data was analyzed across sites to identify overarching themes.

We summarized the workflows across a defined care continuum of lung cancer screening care. LCS workflows were formalized through multiple layers of review by study team members and final member-checking with Advisory Board members at each site.

## Results

**Figure 1.**
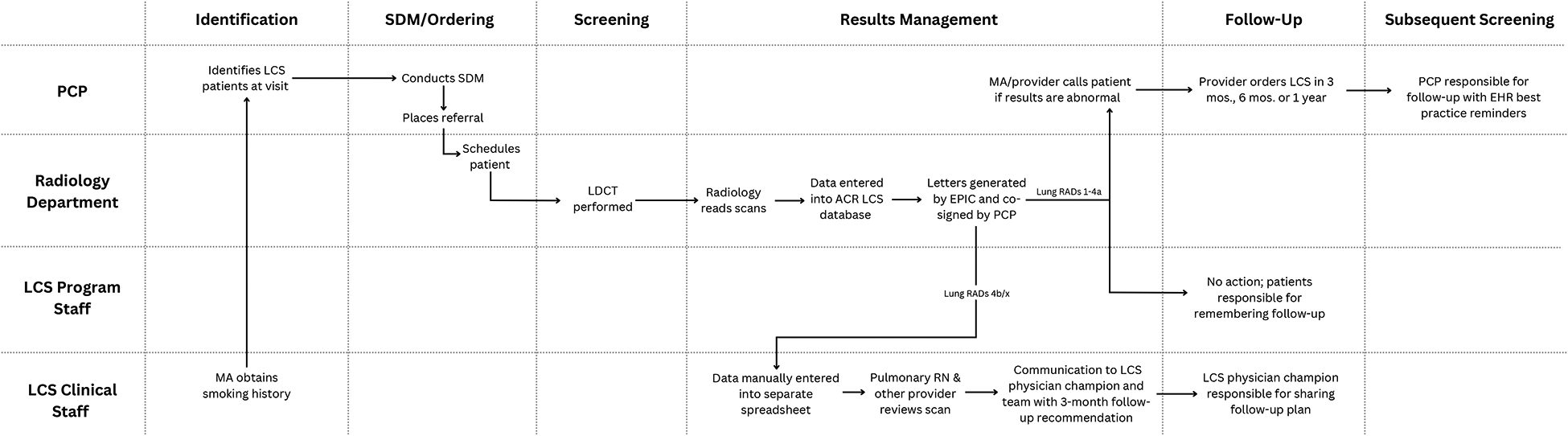
LCS Pre-Intervention Workflow at Confluence Health (Site A). Clinic staff/departments are specified (y axis) with their involvement in the lung cancer screening care continuum (x axis). Abbreviations include: MA (medical assistant), PCP (primary care provider), LCS (lung cancer screening), SDM (shared decision-making), Lung RADs (Lung Imaging Reporting and Data System), RN (registered nurse), EHR (electronic health record).

**Figure 2.**
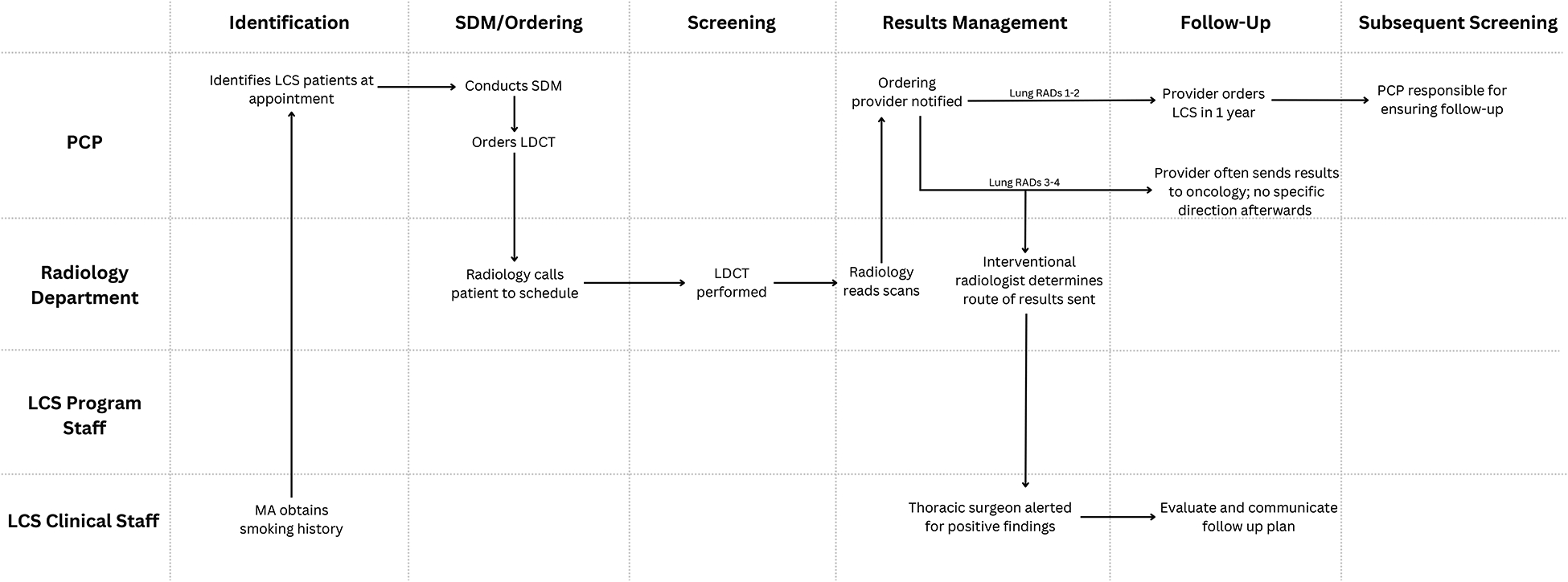
LCS Pre-Intervention Workflow at Skagit Regional Health (Site B). Clinic staff/departments are specified (y axis) by their involvement in the lung cancer screening care continuum (x axis). Abbreviations include: PCP (primary care provider), LCS (lung cancer screening), MA (medical assistant), LDCT (low dose computed tomography).

**Figure 3.**
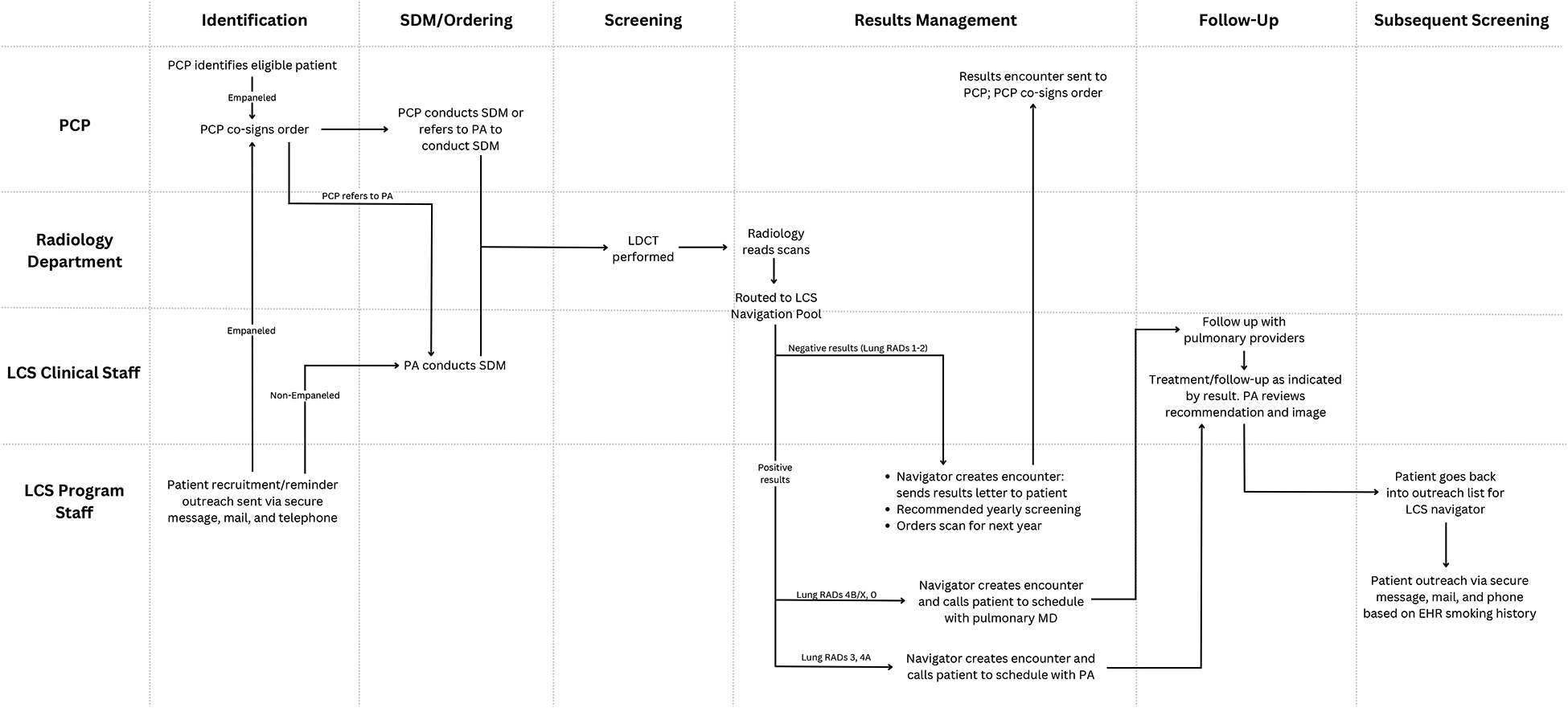
LCS Workflow at The Vancouver Clinic (Site C). Clinic staff/departments are specified (y axis) by their involvement in the lung cancer screening care continuum (x axis). Abbreviations include: PCP (primary care provider), PA (physician assistant), MD (medical doctor), LCS (lung cancer screening), SDM (shared decision-making), Lung RADs (Lung Imaging Reporting and Data System), EHR (electronic health record).

The three community-based programs provide LCS services with variable levels of centralized support. The LCS workflows for each site (Figures 1–3), as described below, demonstrate varied staff involvement along the LCS care continuum. A comparison of key components is presented in Table 1. Key themes about the implementation of LCS from multi-disciplinary providers from both sites (Table 2) include the need for patient education and outreach, provider support and resources, and attention to gaps in care. The need for more patient education and outreach was described to help increase awareness of lung cancer screening and the process of LCS (including engagement in subsequent screenings). Providers also shared needing support and resources, both in terms of electronic tools (e.g., automated reminders, flags) and personnel (e.g., patient navigation) to facilitate LCS. Attention to gaps in care along the LCS continuum was noted as an opportunity for care improvement.

### LCS workflows

#### Site A

At baseline, Site A had a hybrid centralized-decentralized program with an annual volume of ~1900 LCS scans across two LDCT-capable radiology facilities (Figure 1). The LCS program is led informally by a physician champion who is a pulmonologist and the head of the pulmonary practice. The within-system pulmonary medicine department manages all patients with Lung RADS 4 findings. As part of an Accountable Care Organization (ACO), the clinic has a strong focus on preventative care.

Beginning with identification of potentially eligible patients for LCS, the medical assistants within affiliated primary care clinics are largely responsible for collecting patients’ smoking history and recording within Epic.^15^ Tobacco treatment services are not consistently integrated in the LCS continuum and are performed as a PCP responsibility with variable modalities used. A ‘Best Practice Advisory’ will alert providers to discuss LCS among screening-eligible patients. Providers note interest in further use of Epic resources to help facilitate care processes. In addition, case managers identify patients eligible for LCS and other cancer screenings in preparation for their annual wellness visits. Primary care providers (PCPs) can contact the pulmonary nurse navigator to arrange a SDM visit in the pulmonary medicine department or PCPs can themselves complete and document shared decision-making (SDM) and utilize a program-built order set for LDCT. There were no EHR-based tools or program standards for SDM. The referral is transmitted to the radiology department whose schedulers make up to 3 attempts via phone to reach a patient (insurance status is confirmed at the time of scheduling). After the LDCT is performed, the radiologist reads the CT in accordance with the Lung CT Screening Reporting and Data System (Lung-RADS) and publishes the report in the EHR which is transmitted via the “in-basket” function to the ordering provider. The radiology department tracks all LCS scans through a registry submitted to the American College of Radiology. Radiology mails an automated letter to the patient informing them of their results. Ordering providers are responsible for communication of positive results.

Patients/PCPs are primarily responsible for managing annual follow-up scans. For patients with high-risk findings (Lung-RADS 4B/X), a nurse navigator from pulmonology manually enters the patient data into a separate spreadsheet to track follow-up. The LCS physician champion reviews all Lung-RADS 4B/X findings, schedules them to be seen in the pulmonary clinic, and contacts both the PCP and patients to ensure follow-up. For subsequent screenings for patients with Lung RADS 1–4A, the primary care provider orders LCS in 3 months, 6 months or 1 year. For subsequent annual screening, the radiology division will send a LDCT order to the PCP at 11 months, which the PCP must sign to move forward with scheduling.

#### Site B

Site B performs ~1400 LCS scans annually at two LDCT-capable radiology facilities (Figure 2). For identification of screening-eligible patients, the medical assistants in the primary care clinics were trained to collect patients’ history of cigarette smoking, though providers note variability in data capture. Tobacco treatment services are not consistently integrated in the LCS continuum and are performed as a PCP responsibility with variable modalities used. PCPs or other ordering providers perform and document SDM and place LDCT orders. There were no EHR-based tools or decision-support for this process. Several providers within the system had their own notes and templates for such documentation. For LCS-eligible patients, the PCP orders the LDCT and radiology calls the patient to schedule, typically calling up to 3 times prior to closing a referral. PCPs noted the ease of referral and scheduling for LCS. The radiology department reads all LDCTs, and a formal report is posted in the EHR and delivered to the ordering providers’ “In-basket”. The ordering provider is responsible for all result communication, though imaging reports are directly available to patients through the EHR (MyChart feature).^15^ Based on radiology findings, PCPs contact patients with abnormal results and refers them to a specialist (i.e., pulmonary, oncology, thoracic surgery); there was no central nodule program. For annual scans, patients and their PCPs are largely responsible to manage this on their own. In some cases, where there was high concern for lung cancer, the radiologist may independently alert the center thoracic surgeon to review a patient’s record, then determine and communicate a care plan to the patient. These were typically cases of Lung-RADS 4B/X. For negative results, the provider orders LCS in one year with no formalized reminder system. Providers endorsed strong interest in the development of defined care pathways for LCS, noting care pathways and coordination support for breast cancer and colorectal cancer screenings.

#### Site C

The LCS program conducts ~2600 LCS scans annually at one of 2 LDCT-capable sites (Figure 3). The program has robust centralized support throughout the LCS continuum but retains hybrid components to allow involvement by individual PCPs. The dedicated LCS staff includes a pulmonary physician lead, a physician assistant (PA) who is also a certified tobacco treatment specialist (TTS), and a medical assistant/navigator. Tobacco treatment services are integrated throughout the LCS continuum. The PA offers tobacco treatment services to every patient she sees for enrollment.

Medical assistants and other staff within clinics are largely responsible for updating smoking history which informs outreach attempts. To identify and engage potentially eligible patients for LCS, the LCS navigator engages in patient outreach to empaneled patients within primary care clinics via multiple methods including secure electronic message, telephone, and mail. Patients can also schedule directly with a PA on the pulmonology team for an SDM visit. The LCS navigator routinely reviews the PA’s schedule for SDM visits to verify patient eligibility for LCS. In addition, PCPs can identify eligible patients in their panels through the EHR “Care Gaps” module and complete SDM themselves or refer to the PA for an SDM visit. The LCS program PA uses a consistent approach to conduct SDM with a decision aid. Either the PCP or the LCS program PA can order the LDCT.

After the LDCT is performed, the on-site radiology department reads the scans and sends the results to the LCS Navigation pool, where the LCS navigator calls the patient with their results and routes results to the PA and/or PCP. The LCS navigator arranges appropriate follow-up as indicated by the lung RADS score. For abnormal results (Lung-RADs 3’s and 4A’s), the LCS program PA reviews the case. Patients with Lung-RADS 4B/X are directly referred to the pulmonology team by the PA for follow-up and management. The LCS navigator comprehensively tracks and supports all patients for their follow-up care. The LCS navigator tracks patients with abnormal results and calls the patient to ensure that they complete their recommended care and LCS. Patients with negative results are added to the navigator’s annual outreach list. For subsequent screening, the LCS navigator actively tracks and manages an Excel file of all overdue LCS patients, utilizing the report “Overdue Health Maintenance” from an Epic dashboard.^15^ The LCS navigator makes three attempts to reach each patient each year to schedule their annual LCS. If there is no response, patients are kept on the LCS enrollment registry but marked as declined on the EHR “Care Gaps”, which exempts them from outreach for one year.

## Discussion

LCS workflows in three community-based screening programs in Washington state demonstrate varying levels of centralization—or programmatic support--for patients and providers along the LCS continuum. To our knowledge, this is the first case study approach comparing LCS workflows in community-based settings. We suggest that the binary distinction between centralized and decentralized programs fails to capture the reality of actual care pathways, which typically incorporate both centralized and decentralized components and are better understood as existing along a spectrum. Workflows themselves can assist with identifying and categorizing program components by their degree of centralization to help determine areas which may benefit from more central program support. Centralized components within certain phases of the LCS continuum, such as support for tracking when patients are due for follow-up scans and direct outreach to patients with reminders follow-up care, are likely effective approaches to address common challenges with patient adherence.

The workflows demonstrate commonalities and differences as programs deal with challenges to LCS implementation. Prior studies underscore the complexities of LCS processes, and these are reflected in the current workflows. LCS is not as simple as just getting a CT scan. Within each system, providers in various roles -- primary care, specialty care and radiology -- were involved in different aspects of the LCS workflow.

A common challenge for each system to improve uptake of LCS is identifying eligible patients, and all discussed limitations of current systems (largely collection of smoking history by medical assistants at patient visits) to identify screening-eligible patients. This limitation has been reported in the literature, where EHR-based smoking history completion is highly variable and often inaccurate.^20,21^ While no system fully addressed challenges in recording smoking history, Site C’s added layer of directed outreach through electronic and telephone messaging to potentially eligible patients partially addressed this by direct-to-patient discussions of eligibility and screening.

The systems had different approaches to tackling challenges of SDM, which is a required element of LCS ordering by the predominant payor for US screening, the Centers for Medicare and Medicaid Services.^22^ SDM has been described as a provider-level barrier and is time-consuming to LCS uptake. While Site B, a decentralized program, did not provide support for SDM, several providers within the system had their own notes and templates for such documentation. Site A, with a hybrid approach, provided some support through standardized ordering functions and the Care Gap EHR alerts. Site C provided centralized support for this key function, allowing providers to refer to a dedicated provider for SDM or perform it themselves with an order set and note template as part of a smart set that covers the SDM elements.

The centers also took different approaches to LCS follow-up, both in routine annual follow-up and support for patients who had abnormal findings. Site B provided limited support for follow-up annual scans but used internal processes and radiology-to-provider informal referrals for the most concerning findings. Site A used a pulmonary clinic review process to support the most at-risk patients with abnormal findings and tracked and pended orders for follow-up annual scans. Site C had a multi-layered process which included active patient navigation and tracking for those with abnormal findings and direct-to-patient reminders for annual screening. Having dedicated LCS coordination support to manage follow-up care helps to minimize burden for both patients and PCPs.

These granular workflows present different models and illustrate the diversity of programmatic support (beyond just centralized vs. decentralized) offered by screening programs. These workflows also reveal how central support functions may enable enhanced patient support and help to overcome some of the challenges of a complex LCS process. Tobacco treatment services are often overlooked, with missed opportunities to deliver evidence-based tobacco treatment to promote smoking cessation along the LCS continuum.^23^ Centralized programs have consistently been shown to improve LCS care, particularly follow-up. In a comparison of 5 health systems, those screened through a centralized program had twice the adherence to repeat an annual LCS scan as compared to those screened through decentralized programs.^24^ A recent meta-analysis of 9 cohort studies demonstrated that programs with centralized structures, largely consisting of a program coordinator,^25–28^ had an adherence rate of 59% compared to 35% in decentralized programs.^29^ Within a single system with both a decentralized and centralized pathway, adherence to follow-up was higher with the centralized pathway (70% vs. 41%) which employed a care coordinator to manage follow-up.^30^ Centralized elements may also mitigate disparities in screening follow-up^31^, suggesting these components may be most useful to ensure care completion for those with significant health-related social needs.

The strengths of this work include the representation of 3 community-based clinical hubs that provide comprehensive LCS services, and the use of a multi-method data collection approach to ensure the robustness and accuracy of LCS workflows developed for each site. Study limitations include that all clinics were only within Washington state which may limit generalizability to other regions of the United States. While the three included sites vary in their program structures and centralization, they likely do not reflect the diversity of all screening sites across the US. This paper does not include data to assess which of various structures or features are associated with improved LCS outcomes.

In conclusion, LCS programs offer varying degrees of centralized support. These workflows provide a framework for institutions to map their LCS processes and to explore centralized support options which may assist with the implementation challenges of LCS uptake and adherence to follow-up.

## Supplementary Material

Conflict of Interest forms

## Figures and Tables

**Table 1.** Key decentralized and centralized components of lung cancer screening workflows at 3 community sites

	Identification	Tobacco treatment services	Shared decision-making (SDM)/Ordering)	Results Management	Follow-Up	Subsequent Screening
Site A (Confluence Health)	*Decentralized:* Smoking status updated during patient clinic visits.	*Decentralized*: Tobacco treatment services are performed as a PCP responsibility with variable modalities used.	*Decentralized:* PCP* conducts SDM^†^ and places referral for LDCT.^‡^ *Centralized:* Order set support.	*Decentralized*: Referring provider communicates results to patient. *Centralized*: Scan results are entered into the ACR^§^ LCS^ı^ Registry.	*Decentralized*: PCP orders subsequent LCS screenings. *Centralized*: Pulmonary providers and LCS physician champion manage high-risk findings.	*Decentralized*: Patients/PCPs are responsible for remembering follow-up. *Centralized:* EHR^¶^ preventive care reminder.
Site B (Skagit Regional Health)	*Decentralized*: Smoking status updated during patient clinic visits.	*Decentralized*: Tobacco treatment services are performed as a PCP responsibility with variable modalities used.	*Decentralized:* PCP conducts SDM and places referral for LDCT.	*Decentralized*: Referring provider communicates results to patient.	*Decentralized*: PCP orders subsequent LCS screenings. *Centralized*: LCS physician champion manages high-risk findings.	*Decentralized*: Patients/PCPs are responsible for remembering follow-up.
Site C (The Vancouver Clinic)	*Decentralized*: Smoking status updated during patient clinic visits *Centralized:* Program outreach to eligible patients.	*Centralized:* Integrated, multimodal and longitudinal tobacco treatment services are provided through the LCS program PA who is also a certified tobacco treatment specialist.	*Decentralized:* PCP conducts SDM. *Centralized*: PCP refers to the PA^#^ in the pulmonary clinic to conduct SDM and place referral for LDCT.	*Centralized*: Scan results are routed to the LCS Navigation pool with navigator support for follow-up with pulmonology team.	*Centralized*: LCS navigator manages care coordination with pulmonology team.	*Centralized*: LCS navigator manages follow-up and maintains listing of patients for annual outreach.

* PCP: Primary care provider

† SDM: Shared decision-making

‡ LDCT: Low dose computed tomography

§ ACR: American College of Radiology

ı LCS: Lung cancer screening

¶ EHR: Electronic health record

# PA: Physician assistant

**Table 2.** Provider perspectives about the implementation of lung cancer screening (n=15)

Key theme	Supporting quotations
Need for patient education and outreach	“You know breast cancer screening, colon cancer screening has had so much good patient education campaigns. Um. And I do think that maybe that’s something that has been missing with lung cancer screening is that education and outreach.” [Site A] “Sharing information and I think we also need some information in terms of, you know, sharing that information and the need, uh, in terms of the frequency of monitoring when you have a positive result, not necessarily cancer, but nodules.” [Site B]
Provider support and resources	“And if it’s normal they get a recall like in one year, if you can have that automated for lung cancer screening, that will be, I mean, less, you know, a burden, you know, for the physician.” [Site B] “So definitely having another person to sort of like holding that patient accountable, you know, or help them navigate, you know, especially if you have a finding, uh, I think it’s very— it will be very beneficial.” [Site B] “I think it it’s a matter of everything is put on the primary care doc’s plate. And so, it gets to be very crowded and sometimes your vision gets a little tunneled with other things that happen. It just, it’s just we need to find a better system to try and do this preventative care stuff.” [Site A]
Attention to gaps in care	“I do think, you know, making sure it happens annually is, is problematic. It does get flagged, but if a patient doesn’t come in, you can definitely have gaps in that.” [Site A] “Probably one of the challenges is where if we need to get pulmonary involved in the process… And so, I think that’s one of the probably, you know, challenges or barrier to, uh, streamline, you know the referral process.” [Site B]

## Data Availability

De-identified qualitative data and summarized datasets created during the current study will be made available from the corresponding author on reasonable request.
